# Generation and clinical potential of functional T lymphocytes from gene-edited pluripotent stem cells

**DOI:** 10.1186/s40164-022-00285-y

**Published:** 2022-05-14

**Authors:** Rongqun Guo, Wei Li, Yadan Li, Yingmei Li, Zhongxing Jiang, Yongping Song

**Affiliations:** 1 Department of Hematology, The First Affiliated Hospital of Zhengzhou University, Zhengzhou, 450052 Henan China; 2 Academy of Medical Science, Henan Medical College of Zhengzhou University, Zhengzhou, 450052 Henan China

**Keywords:** T-cell generation, Pluripotent stem cells, Conventional T cells, Engineered T cells, Gene editing

## Abstract

Engineered T cells have been shown to be highly effective in cancer immunotherapy, although T cell exhaustion presents a challenge for their long-term function. Additional T-cell sources must be exploited to broaden the application of engineered T cells for immune defense and reconstitution. Unlimited sources of pluripotent stem cells (PSCs) have provided a potential opportunity to generate precise-engineered therapeutic induced T (iT) cells. Single-cell transcriptome analysis of PSC-derived induced hematopoietic stem and progenitor cells (iHSPC)/iT identified the developmental pathways and possibilities of generating functional T cell from PSCs. To date, the PSC-to-iT platforms encounter several problems, including low efficiency of conventional T subset specification, limited functional potential, and restrictions on large-scale application, because of the absence of a thymus-like organized microenvironment. The updated PSC-to-iT platforms, such as the three-dimensional (3D) artificial thymic organoid (ATO) co-culture system and Runx1/Hoxa9-enforced iT lymphopoiesis, provide fresh perspectives for coordinating culture conditions and transcription factors, which may greatly improve the efficiency of T-cell generation greatly. In addition, the improved PSC-to-iT platform coordinating gene editing technologies will provide various functional engineered unconventional or conventional T cells. Furthermore, the clinical applications of PSC-derived immune cells are accelerating from bench to bedside.

## Introduction

Embryonic stem cells (ESCs) isolated from blastocytes can be cultured in vitro and used to generate engineered cells and animal models. Furthermore, the technology of reprogramming somatic cells to induced pluripotent stem cells (iPSCs) [1, 2] provides a possible way to explore the applications of stem cells in regenerative medicine, without ethical and immune rejection concerns [3]. A recent study showed that the application of clinical-grade iPSC-derived functional retinal pigment epithelium is feasible and safe [4]. However, the biggest challenge is the inefficient reconstitution of iPSC-derived phenotypic cells in vivo.

Edited T cells are being studied for engineering chimeric antigen receptor (CAR) T cells which are a form of major cellular therapy for hematological malignancies [5–7]. However, the application of cell immunotherapy is limited by the availability of autologous T cells and associated complications and resistance [8–10]. Meanwhile, the tumor-killing ability of patient-derived engineered T cells is suppressed by the senescent and exhausted T compartments, or by increasing the Treg subset [11, 12]. Compared with rare HSPCs, PSCs have unique advantages, such as their efficient gene editing and long-term self-renewal properties in vitro. These advantages make them the best candidates for T-cell generation. However, the progress of PSC-to-iT technology is still facing hurdles because the thymic niche cannot be accurately simulated in vitro. With these challenges, another straightforward approach is to use the in vivo microenvironment to educate PSC-derived thymus-seeding progenitors (TSPs). In this review, we describe recent progress in understanding T cell development in the thymus, single-cell transcriptomes of PSC-iHSPC/iT, PSC-based T lymphocyte generation, and the potential applications of gene editing in the PSC-to-iT platform.

## T lymphopoiesis in the embryo and adult

**Fig. 1:**
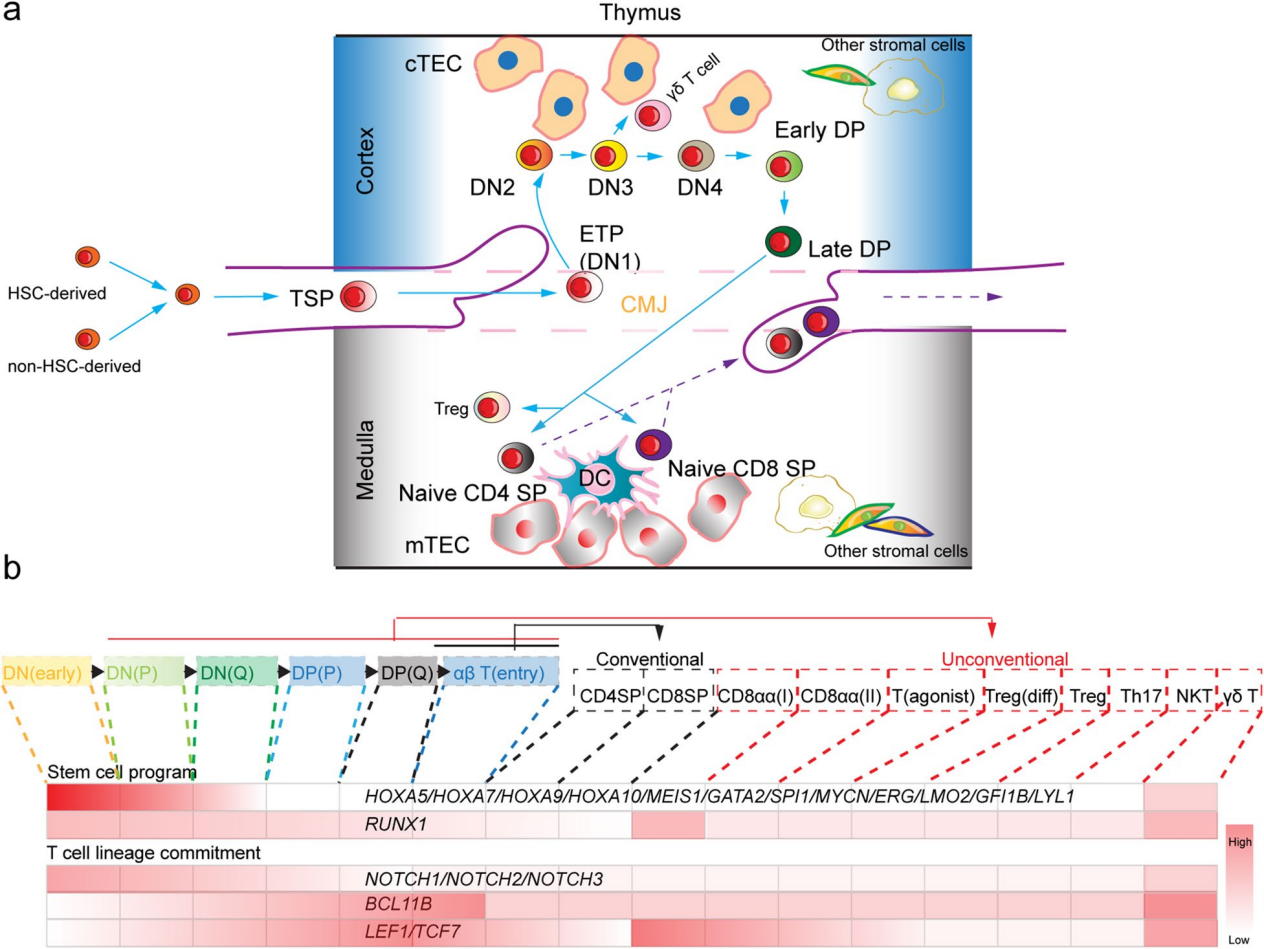
T lymphopoiesis in thymus.** a** HSC or embryonic non-HSC-derived TSP migrate into the CMJ, then differentiate into DN and DP cells, which are educated by different stromal cells (for example cTEC) in the cortex. DP cells mature into naïve conventional T cells and unconventional T cells in the presence of stromal cells (such as mTEC and DCs) in the medulla. **b** Different stages in T lymphopoiesis. A new cell atlas of human thymic development showed a unique pattern of T lymphopoiesis [31]: DN (early) → DN (P) → DN(Q) → DP(P) → DP(Q) → αβ T (entry), and new unconventional T subsets. Different TFs regulatory networks drive the formation of different stages and subsets. *DN* double negative T cells, *DP* double positive T cells, *P* proliferation, *Q* quiescent, *HSC* Hematopoietic stem cells, TSP thymus-seeding progenitors, *CMJ* cortico-medullary junction

T lymphocytes play an essential role in adaptive immunity, including pathogen elimination [13], host homeostasis [14], and anti-tumor activity [15]. During hematopoiesis, fetal liver [16] and bone marrow-derived hematopoietic stem cells (HSCs) [17] differentiate into TSP, such as lymphoid-primed multipotent progenitors (LMPP) [18]. Current evidence demonstrates that non-HSC-derived TSP supports T lymphopoiesis before the emerge of HSCs [19–23] (Fig. 1a). Particularly, Flt3 signals induce *CCR9* expression in TSP [24], which is then recruited into the thymus through *CCL25* (*TECK)*, secreted by thymic epithelial cells (TEC), and recruits the TSP into the thymus [25].

Once the TSP cells seed into the cortico-medullary junction (CMJ) in the thymus [26], a new identity is acquired as early thymic progenitors (ETP) [27]. Published studies have shown that ETP have lineage pattern similar to that of LMPP with T cells, B cells, NK cells, and myeloid potential [28–30]. And ETP lack the megakaryocytic and erythroid potential. As a specialized organ for T lymphopoiesis, the thymus provides a complex, highly ordered, and unique niche. Although the microenvironment is complex, the thymus provides cascaded and conserved signaling pathways such as Notch signaling, morphogenic pathway, and protein tyrosine kinase signaling. The thymus mainly contains hematopoietic cells (T cells, B cells, NK cells, monocytes, dendritic cells [DCs], and macrophages) and non-hematopoietic stromal elements (TEC, fibroblasts, vascular smooth muscle cells, lymphatic endothelial cells, and endothelial cells) [31]. Nude mouse research has shown that TEC is the most pivotal element [32–34]. TEC and other stromal cells foster natural T lymphopoiesis by providing a microenvironment and expressing chemokines, Notch ligands (JAG1, JAG2, Delta-like ligand 1, and 4), Wnt ligands, Hh proteins, BMPs, SCF, and IL7 [35–52]. Coordinating with stem/progenitor-cell gene network (*Hoxa9*, *Runx1*, *Gata2*, *Meis1*, *Lmo2*, *Myb*, *Mycn*, and others) (Fig. 1b), the thymic niche-provided Notch signal initiates the T-lineage-specific development program of TSP, as a pre-commitment phase [53]. Also, interfering with the function of Notch results in a complete block of T lymphopoiesis [54, 55].

During T lymphopoiesis in the thymus, ETP differentiates into immature CD4/CD8 double-negative thymocytes, (DN1, DN2, DN3, and DN4 cells), and immature CD4/CD8 double-positive thymocytes (DP cells) (Fig. 1a). After undergoing positive and negative selection, DP cells mature into naïve mature CD4 single-positive (CD4SP) thymocytes and CD8 single-positive (CD8SP) thymocytes. In addition to conventional T cell subpopulations (TCRαβ^+^ CD4SP T subset and TCRαβ^+^ CD8SP T subset), TSP also differentiate into unconventional T cell subpopulations (γδ T subset, Treg subset, CD8αα^+^ T cell subset, natural killer T-like [NKT-like] subset, and fetal TH17-like subset) [31]. The rapid deployment of single-cell sequencing technology has helped us to discriminate rare T precursors and unconventional T subsets from the thymus atlas [31], analyze the dynamics of thymocyte development, and simulate T-cell generation in vitro. New unconventional subsets were identified through single-cell RNA sequencing (sc-RNAseq), such as the CD8αα(I) subset (expressing *PD-1*, *TNFRSF9*, *CD72*, *CREB3L3*, *GNG4*, and *XCL1* at mRNA level), CD8αα(II) subset (*PD-1*, *ZNF683*, and *MME*), T (agonist) subset (*MIR155H*), Treg (diff) subset (*IKZF4*, *GNG8*, and *PTGIR*), and Th17-like cell subset (*CD40LG*, *RORC*, *KLRB1*, and *ZBTB16*) (Fig. 1b). The marker genes *CD34*, *ST18* and *IGLL1* were used to identify cells at the double-negative (DN) early stage.

## Single-cell transcriptome analysis of PSC-iHSPC/iT

**Fig. 2:**
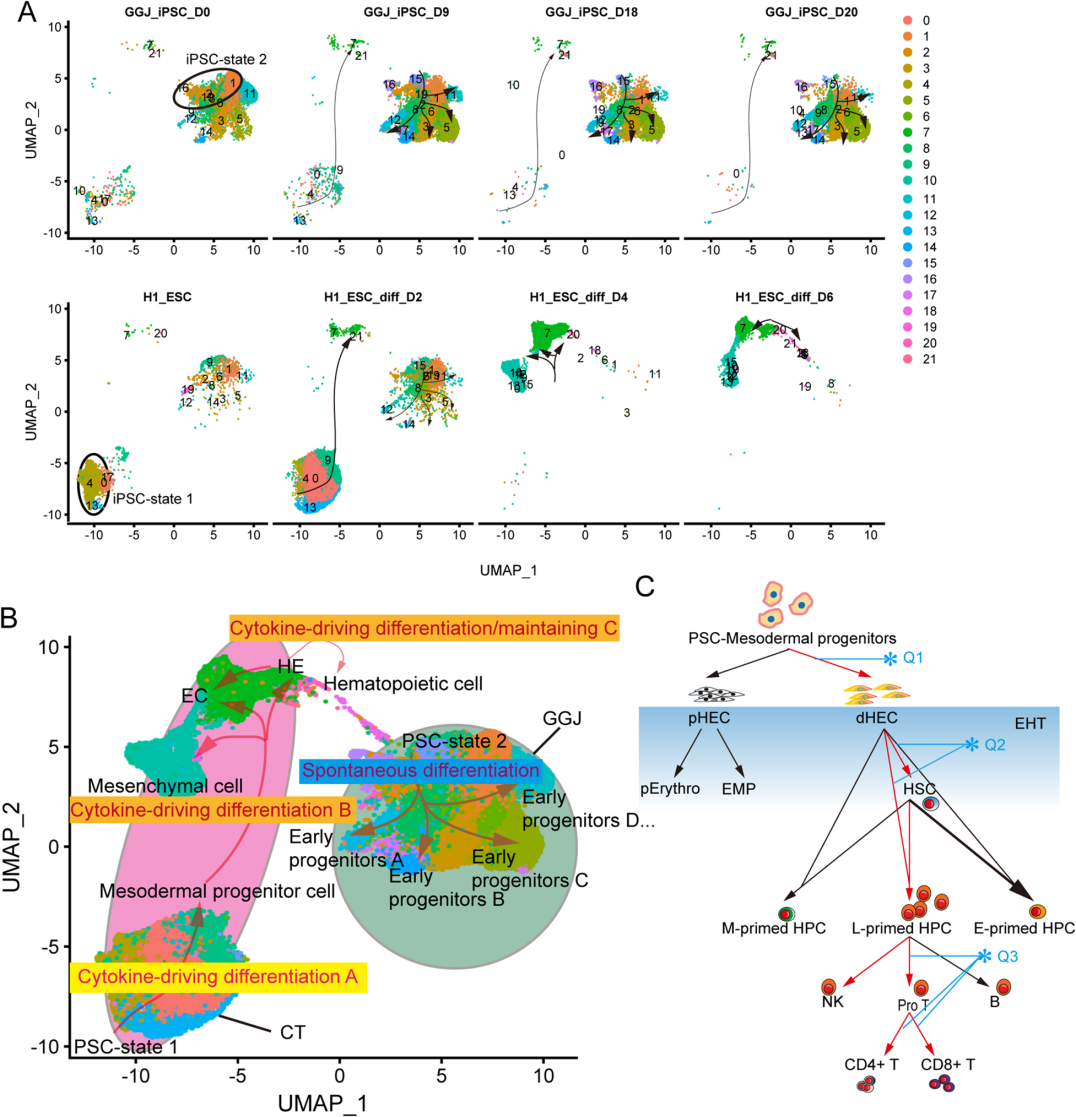
scRNA-seq technology reveals the heterogeneity of PSCs and its derivatives and the complexity of the hematopoietic differentiation process of PSCs.** a** UMAP on the transcriptome of the PSCs and PSC-derived cells from Cheng’s lab (H1 ESC and H1 ESC-derived cells at day 2/4/6 during directed hematopoietic differentiation) and Guo’s lab (iPSCs and iPSC-derived EB cells at day 9/18/20 without adding any lineage-specific cytokines or conditions).** b** Schematic diagram of two types of PSC differentiation with or without lineage-specific cytokine combinations based on scRNA-seq datasets of Cheng’s lab and Guo’s lab. **c** A brief schematic overview of key differentiation steps (Q1, Q2, and Q3) from PSCs to T lymphocytes. *pHEC* primitive HEC, pErythro primitive erythroid cells, EMP erythromyeloid progenitor, *dHEC* definitive HEC, *M-primed HPC* myeloid-primed HPC, *L-primed* HPC lymphoid-primed HPC, *MEP* megakaryocyte-erythroid progenitor, *Pro T* progenitor T-cells

Single-cell transcriptional profiling has been used for the analysis of adult/embryonic hematopoietic development and immune states monitoring. In most cases, it is difficult to accurately confirm whether the PSC-derived cells are the desired cell types. Indeed, the desired PSC-derived cells were mixed with undifferentiated PSC, mesodermal progenitors, endothelial cells, lineage-specific hematopoietic progenitors, and other unexpected cell types. Fortunately, the single-cell transcriptional sequencing technology has made it possible to reliably delineate the directed differentiation process of PSC to hematopoietic lineages (Table 1). Using such technology, Guo lab reported that PSCs are heterogenous cell populations themselves and thereby have variable efficiency of hematopoietic differentiation [56]. The PSC cell lines from different labs also showed obvious differences identified through sc-RNAseq analysis (Fig. 2a). Without mesodermal lineage differentiation-related cytokines, it is difficult for the PSCs to generate hemogenic endothelium cells (HECs) (Fig. 2a). The combination of glycogen synthase kinase (GSK) 3 inhibitor (CHIR99021) with BMP4 and Activin A helps PSCs efficiently differentiate into mesoderm progenitors (cytokine-driving differentiation pattern A). VEGF and bFGF further enforce these progenitors to differentiate into EC and HECs/hematopoietic cells (cytokine-driving differentiation pattern B) (Fig. 2b). There is strong evidence indicating that the heterogeneities of embryonic and PSC-derived HECs result in diverse lineage potentials as demonstrated at the sc-RNAseq levels (Fig. 2c)[57–60]. Based on the decision of the hematopoietic fate, HECs can be divided into two groups: primitive hematopoietic development-related HECs (pHECs) and definitive HECs (dHECs); dHECs are major populations producing T-lineage cells. Despite the comprehensive work of embryonic hematopoietic development at the sc-RNAseq level, mimicking hematopoietic development using PSC-derived hematopoietic cells remains a challenge. With the help of embryonic hematopoietic development and adult hematopoiesis at sc-RNAseq levels, the hematopoietic differentiation of PSC is moving closer and closer to physiological hematopoietic development by adding missing critical transcription factors and culture niche [60, 61]. Unlimited functional PSC-derived HSC or T/NK cells are one of the ultimate goals of PSC-based regenerative medicine, and several problems remain to be solved, such as Q1: how to efficiently get dHECs but not pHECs; Q2: how to enforce the differentiation of dHEC into *bona fide* HSCs and lymphoid-primed HPCs; and Q3: how to provide a suitable niche for T-lineage to mature and harvest both functional CD4^+^ T and CD8^+^ T cells robustly. Taken together, the single-cell transcriptional profiling of PSC-derived cells clearly shows the possibility of generating functional T cells in vitro, although some problems still remain.

**Table 1:** Single-cell transcriptome datasets of PSC-derived cells during hematopoietic differentiation

Year of publication	Cell types	Dataset ID (data type)	Generating T cell or not (Condition)	Function
2017 [62]	hESC-derived HE, non-HE, and HP cell populations	-(scRNA-seq)	Unknown	Unknown
2017	hiPSC-derived CD34 + cells	GSE87422 (single cell qRT-PCR)	Unknown	Unknown
2020 [63]	CD34 + CD43- derived cells	E-MTAB-8205 (scRNA-seq)	Unknown	Unknown
2020 [64]	CD235a-CD43 + cells	https://lab.antonellafidanza.com (scRNA-seq)	Unknown	Unknown
2020 [65]	iPSC-derived EB at day 9/18/20	GSE134355 (scRNA-seq)	Unknown	Unknown
2020 [63]	iPSC-derived CD34 + /CD43 − cells and their derivatives	E-MTAB-8205 (scRNA-seq)	Unknown	Unknown
2020 [66]	hESCs-derived CD43 + HPCs	GSE148215 (scRNA-seq)	CD8^+^ T/CD4^+^ T/CD4^+^CD8^+^ T (OP9-DLL4 co-culture)	Unknown
2021 [67]	hPSC/hPSC-derived D2/4/6 cells	GSE145859 (scRNA-seq)	CD3 + T (ATO); CD8 + T(anti-CD3/28 stimulation)	Cytotoxic function in vivo
2021 [68]	CD45 + CD34 + CD7 + iPSC-proT cells	GSE169279 (scRNA-seq)	CD3 + αβ T cells (DL4-μbeads)	Unknown

*iPSC* induced pluripotent stem cells, *HPSC* hematopoietic stem cells, *scRNA-seq*  single-cell RNA sequencing; HESC; EB; *ATO* artificial thymic organoid, *qRT-PCR* quantitative real-time-polymerase chain reaction

## Generation of T lymphocytes from PSCs in vitro

Reprogramming of somatic cells to iPSCs [1] provides the possibility of solving the source problem arising from limited T-cell or HSC sources [69]. Early studies have illustrated the ability of ESCs differentiating to T lineage *in vitr*o and in vivo [70–75]. Based on the understanding of T-lineage commitment in the thymus, researchers have established OP9-DLL1 as stromal cells to harvest T-lineage commitment cells from PSCs [76]. The OP9-DLL1/4-PSC co-culture system is widely applied to T cell development research in vitro as a stable and efficient culture method [77–81]. Interestingly, OP9-DLL1/4-PSC coculture exhibited unconventional T-subset bias in vitro, such as γδ T cells and NKT cells, compared with T lymphopoiesis in the thymus in vivo [77, 78].

**Fig. 3:**
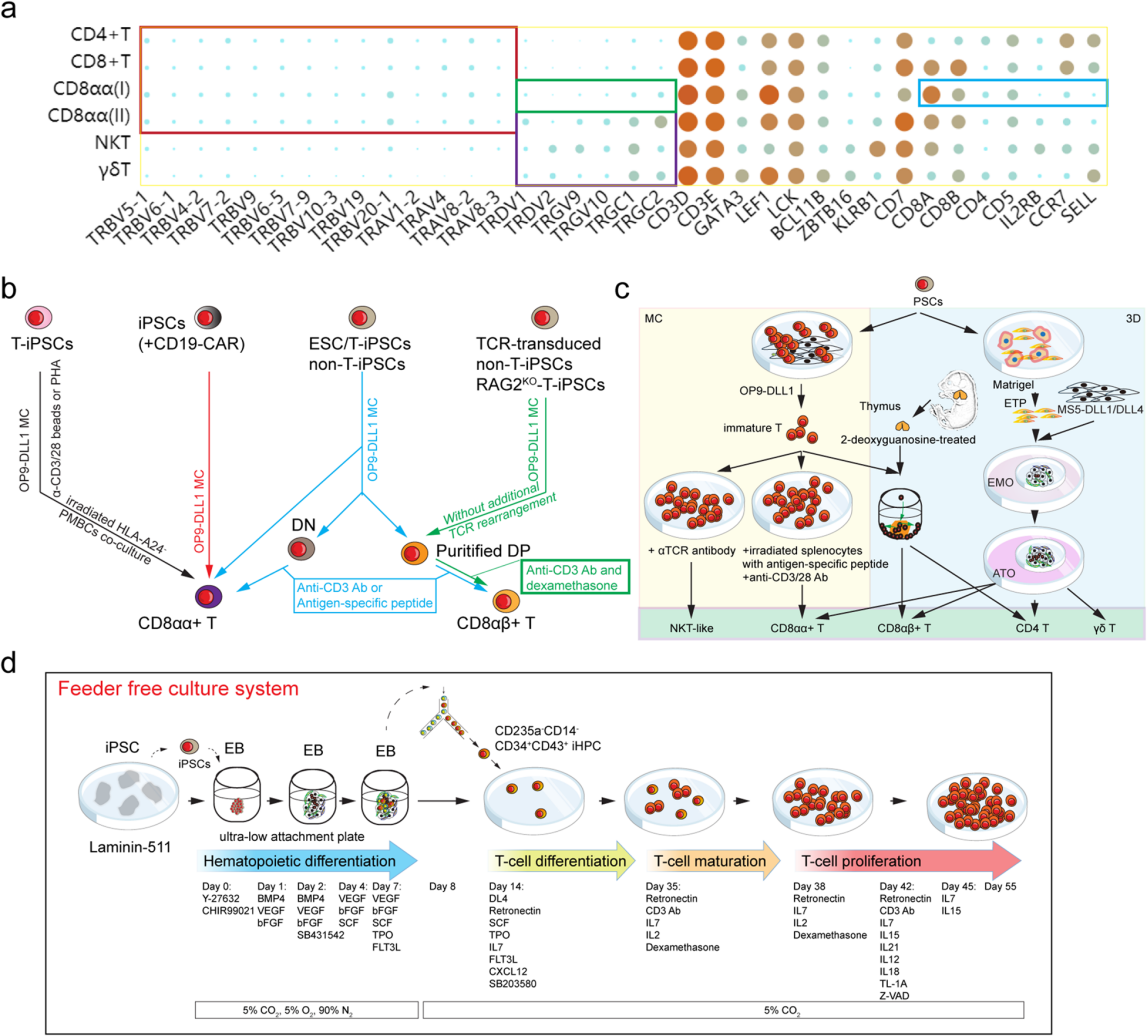
Schematic diagram of the differentiation strategies to generate T lineage subsets from PSCs.** a** iPSC-derived “γδ-like” CD19-CAR-T cell [82] is similar to the GNG4^+^CD8αα^+^ T(I) subset, which identified the phenotype of TCRαβ^+^TCRγδ^−^CD8α^+^CD8β^−/low^IL2RB^−^CCR7^−^CD62L(SELL)^−^ from the cell atlas of the human thymic development [31] (https://developmentcellatlas.ncl.ac.uk/datasets/HCA_thymus/fetal_thymus_Tcell_interactive_gene_expression_heatmap.html).** b** T-cell generation models in OP9-DLL1/PSC monolayer co-culture system. The ectopic-expressing Notch ligands on stromal cells enhanced the T-lineage commitment, the following immature T cells mature into CD8αα^+^ T cells or CD8αβ^+^ T cells under different culture conditions.** c** T-cell generation models in 3D co-culture system. 2-deoxyguanosine-treated thymus lobes, and MS5-DLL1/4-constructed ATO, can be used for 3D coculture system, which may provide a thymus-like microenvironment. MC, monolayer culture. **d** A scalable iPSC-to-iT platform under Ff condition

The function of PSC-derived T lymphocytes was only partially defined, because of the random TCR rearrangements during T lineage differentiation in vitro. Meanwhile, complicated and unpredicted T-lineage commitment in vitro limits the knowledge about whether HLA restriction or positive/negative selection is normal [82]. The use of antigen-specific CD8^+^ T-derived iPSCs to regenerate specific T cells is a promising source of off-the-shelf immune cells [83]. However, endogenous expression of *RAG1* and *RAG2* may lead to an undesirable loss of antigen specificity with TCR rearrangement [69]. As a classic example of cellular immunotherapy [84], anti-CD19 CAR (CD19-CAR)‐modified T‐cell therapy provides new ideas for antigen-specific T-cell generation. One study showed the potential of anti-tumor therapeutic CAR-engineered PSCs [82]. Intriguingly, CD19-CAR engineered T cells from iPSCs were innate “γδ-like” CAR-T cells instead of conventional T subsets. Single-cell sequencing technology provides an opportunity to understand rare and unconventional cell subsets. Multiple-development-stage, large-scale, and high-throughput sc-RNAseq analysis of the human thymus revealed a rational framework for the generation of functional T lymphocytes [31]. The iPSC-derived “γδ-like” CD19-CAR-T cells conform the phenotype of TCRαβ^+^TCRγδ^−^CD8α^+^CD8β^−/low^IL2RB^−^CCR7^−^CD62L (SELL)^−^ (Fig. 3a). CD8αβ heterodimers, not CD8αα, provide co-receptor function for CD8-dependent TCR, as an effective co-receptor for TCR signaling [85] and binding to MHC-I molecules efficiently [86].

Following a previous OP9-DLL1/PSC monolayer co-culture protocol [87], Takuya Maeda and his colleagues harvested PSC-derived LMP2-specific CD8αα^+^ T cells, with low cytotoxic activity compared with primary CTLs [88] (Fig. 3b). Interestingly, purified iPSC-derived DP cells, but not DN cells, could differentiate into CD8αβ T cells after stimulation with CD3 Ab or agonist peptide (Fig. 3b). To avoid the loss of antigen-specificity caused by TCRα rearrangement, Shin Kaneko’s lab depleted *RAG2* by CRISPR-Cas9 in antigen-specificT-derived iPSCs (T-iPSCs) [89]. Alternatively, myeloid cell-derived iPSCs carrying TCR expression cassettes have overcome the hurdle of mispaired TCRαβ. Song et al. also established a solid protocol for PSC-to-iT based on the OP9-DLL1/PSC monolayer co-culture system, which helped to harvest functional hepatitis B virus (HBV) Ag-specific T lymphocytes and target HBV Ag^+^ cells in a mouse model [90]. In summary, without a well-organized thymus-like microenvironment, the designed program of T lineage from PSCs is disrupted by unpredictable factors, such as PSC-derived unfavorable cells, abnormal TCR signal, or endogenous *RAG* gene expression.

A recent study compared the OP9-DLL1/PSC monolayer co-culture with 3D thymic co-culture and identified aberrant physiological developmental signals of T development in the OP9-DLL1 monolayer [91] (Fig. 3c). After agonist peptide and anti-CD3/CD28 stimulation, PSC-derived CD8β T cells with weak immunophenotype, converted characteristics as CD8αα^+^/DN cells in the OP9-DLL1/PSC co-culture system, and anti-TCR antibody stimulation leading to NKT-like cells separately. To generate an in vitro physiological thymic microenvironment, a fetal thymic organ culture (FTOC) system was used to facilitate the maturation of iPSC-derived immature T cells to CD8αβ T cells. As designed, 2-deoxyguanosine-treated fetal thymic lobes enforced the generation of functional CD62L^+^CD69^−^MHC-I^+^ CD8αβ T cells.

Although the 3D thymic co-culture system has unique advantages, the source of primary organs, production expansion, and quality control are irreconcilable challenges. The strategy of the 3D ATO co-culture system ensures positive selection and harvests conventional T cells from HSPCs in vitro, which provides a new method for conventional T-subset generation [92]. Crooks extended the ATO strategy to the PSC-to-iT field [93] (Fig. 3c). Purified PSC-derived CD326^−^CD56^+^ embryonic mesodermal progenitors (EMPs) were aggregated into 3D embryonic mesodermal organoids (EMO) with MS5-DLL1/4 in low-serum conditions. After two weeks, the T-lineage commitment medium was used for ATO culture where derivation of PSCs produced a dominant CD8αβ T subset with transit CD8αα T subset and a few CD4SP T cells. The pattern of *CDR3* lengths and *DNTT* expression indicated that PSC-ATO could provide a fetal thymus-like microenvironment. By applying the same strategy, Shin Kaneko’s lab could also harvest CD4^+^ T helper (Th) cells with Th1 or Th2 function mediated by knocking out *IL4* or *TBX21*, respectively [94]. Altogether, the FTOC system and ATO systems provide CD8αβ^+^TCRαβ^+^ T-cell and CD4^+^ Th cell generation platforms, which are closer to the thymic microenvironment. However, these approaches must be optimized to save time, reduce complex steps, and become operation friendly.

Shin Kaneko’s lab developed an efficient and scalable feeder-free (Ff) differentiation system that can regenerate cytotoxic T-cells from iPSCs[95]. This Ff system drives a well-defined T lineage commitment in vitro: iPSCs → CD235a^−^CD14^−^CD34^+^CD43^+^ iHPCs → CD7^+^CD5^+^ T-cell progenitors → CD4^+^CD8αβ^+^ DP cells → CD8αβ iT cells. The combination of several factors (CXCL12, SB203580, retronectin, IL-7, IL-15, IL-12, IL-18, IL-21, TL1A, and so on) in synergy helps to establish a novel strategy of large-scale production of CD8αβ^+^ T cells from iPSCs (Fig. 3d). Notably, CXCL12 and SB203580 can expand iT by approximately 3000-fold during T-cell differentiation. This culture system could avoid safety issues, such as replacing OP9-DLL4 stromal cells with DLL4 protein, FBS with BIT (BSA supplemented with insulin and transferrin) or serum-free medium. This is a credible and comprehensive culture system of PSC-to-iT; however, reducing the tedious technical process will be a serious challenge.

## Reconstitution of T lymphopoiesis from PSCs in vivo

### Reconstitution of T lymphopoiesis from PSC-derived TSP

Obtaining engraftable functional PSC-derived mature lineage cells is the most important challenge in the field of regenerative medicine, owing to the challenges of the recipient's immunological rejection, dysfunctional cell survival/ proliferation/differentiation signal, or inability of the cells to migrate to a suitable microenvironment [76, 80]. PSC-derived cells cannot effectively exert their physiological functions in vivo. However, under specific circumstances, PSC-derived T progenitors can produce CD4SP T cells and CD8SP T cells in subcutaneously implanted FTOCs, which indicates that these T progenitors lack thymus-seeding ability [76]. The latest platform of physiological conventional T-subset generation in vivo provides a novel idea for the practical application of PSC-to-T technology [60, 96].

**Fig. 4:**
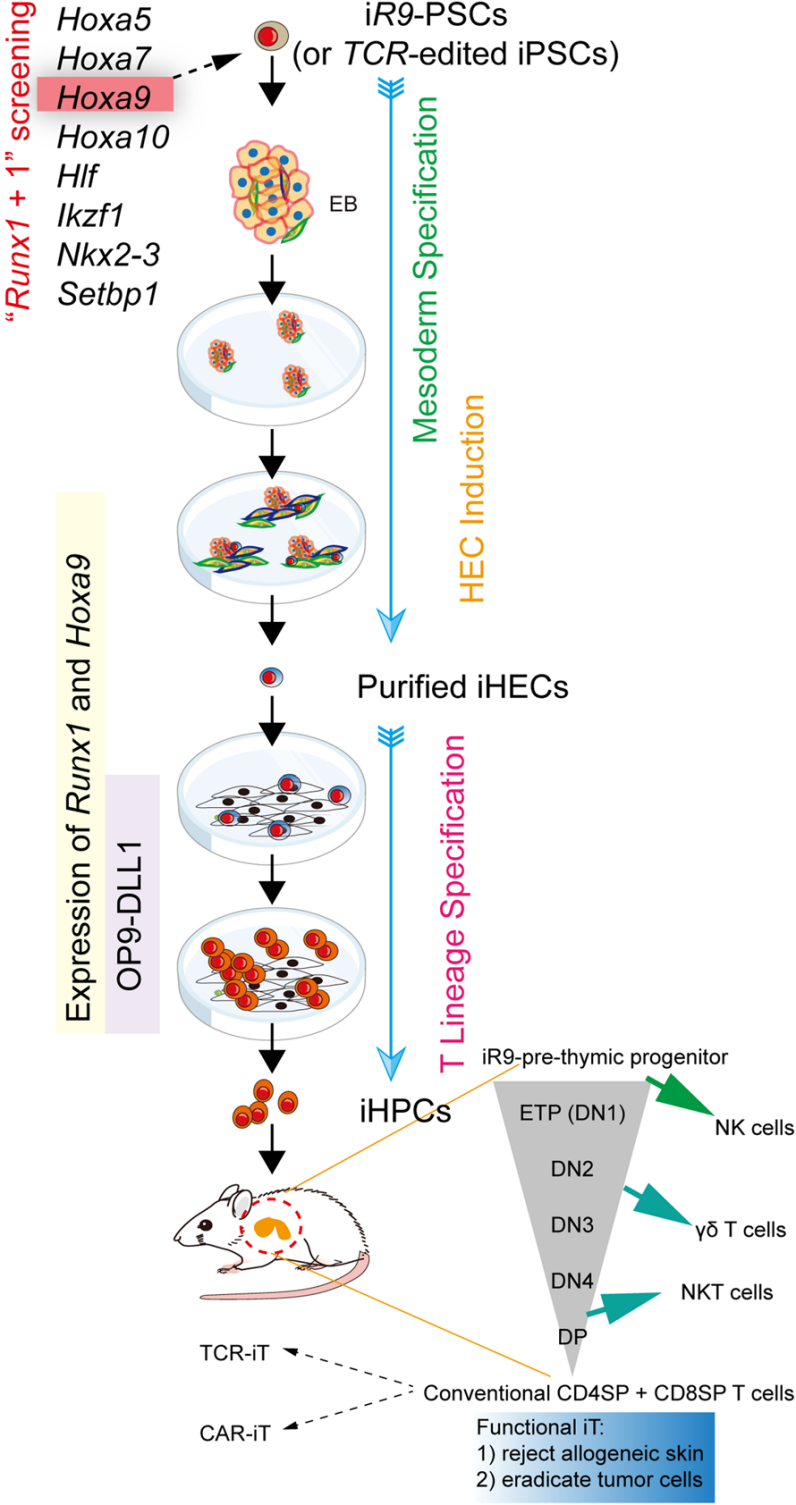
Summary of the reconstitution of T lymphopoiesis in vivo by transient *Runx1* and *Hoxa9* expression. Specific TFs combination of *Runx1* and *Hoxa9* were screened from several important TFs, which robustly drive the T-lineage specification. The i*R9*-PSCs differentiate into iHECs by mesoderm specification and HEC induction. And the OP9-DLL1 stomal cells promote T-lineage commitment with the transit expression of *Runx1* and *Hoxa9*. PSC-derived iHPCs can be transplanted into B-NDG mice for T lymphopoiesis in the thymus, and differentiate into different functional T subsets, as the classical T-cell developmental pattern

Transcription factors (TFs) are the core organizers of cell fate [97, 98]. Among them, *Runx1* is the master regulator of embryonic hematopoietic development [99, 100], This factor helps the generation of T cells from PSCs. Transient expression of *Runx1* during hematopoietic commitment, enforced the emergence of pre-HSC-like (CD31^+^CD41^low^CD45^−^c-Kit^+^CD201^high^) inducible hemogenic endothelial cells (iHECs) and HPC-like cells, but not T cells in vitro or in vivo in the further differentiation, indicating that *Runx1* alone is not sufficient to initiate the PSC-to-iT program. Further scRNA-seq analysis showed that inducible *Runx1*-mESC-derived iHEC has divergent gene expression patterns when compared with those from mouse E11 Type I pre-HSC (T1-pre-HSC), especially missing the expression of some important hematopoietic TFs, such as *Hoxa* family members, *Hlf*, *Ikzf1*, *Setbp1*, and *Nkx2-3*. Using the strategy of “*Runx1* + 1”, the combination of *Runx1* and *Hoxa9* can enforce strong T lineage commitment markedly, but not other combinations (Fig. 4). The Hoxa family is essential for the proliferation of HSPC and lymphoid commitment, especially Hoxa9 [101]. The inducible *Runx1*-*p2a*-*Hoxa9* mESC (i*R9*-ESC)-derived iHEC showed molecular features between E11 EC and T1-pre-HSC, and then differentiated to TSP-like (Lin^−^c-kit^+^CD127^+^/CD135^+^) progenitors. Also i*R9*-ESC-derived iHECs gave rise to T cells at the single-cell level efficiently, regardless of in vitro or in vivo conditions. After i*R9*-ESC-derived pre-thymic progenitors were transplanted into irradiated B-NDG mice, these progenitors generated inducible T (iT) cells, which showed features of abundant TCR diversity, multi-organ distribution, and conventional T development pattern. More importantly, different stages (DN1, DN2, DN3, DN4, DP, and conventional SP) of T lymphopoiesis were detected in the thymus. These PSC-derived iT cells have a physiological adaptive immune response, which has been identified by allogeneic skin transplantation. *TCR*-edited iPSC-derived iT cells efficiently eradicated E.G7-OVA tumor cells. Furthermore, these iPSC-derived functional iT cells can be further engineered with CD19-CAR T cells, which can robustly eliminate lymphoma cells both in vitro and in vivo [102]. Combining this strategy with those of Notarangelo’s lab or Mikkers’ lab reconstituted T lymphopoiesis in vivo and rescue severe combined immune deficiency (SCID) patients early in life [103, 104]. Altogether, regenerated *bona fide* TSP-like cells through transient expression of *Runx1* and *Hoxa9* are effective, allow normal conventional T development in the thymus, and avoid the generation of abnormal cells because of in vitro unfavorable factors.

### Reconstitution of T lymphopoiesis from PSC-derived induced HSC

**Fig. 5:**
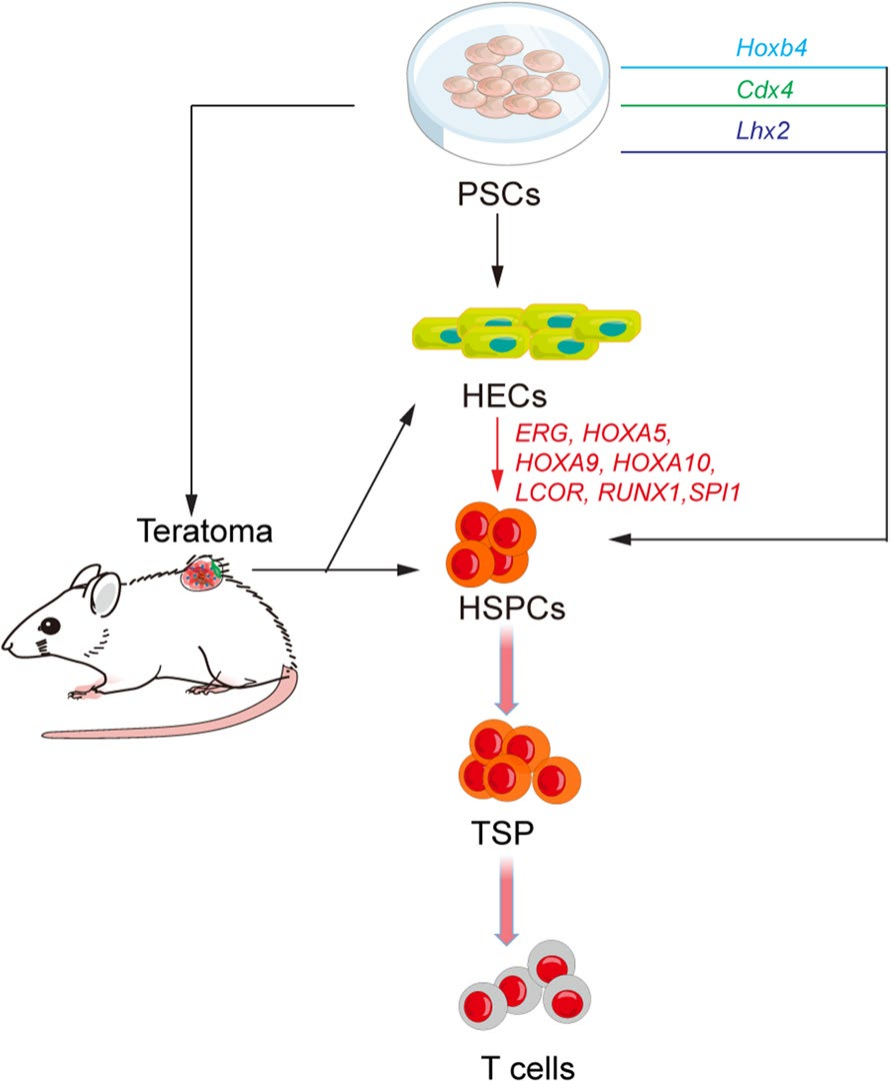
The strategies of reconstitution of T lymphopoiesis in vivo by iHSCs from PSCs. Different TFs can be used to drive the iHSPCs formation and teratoma can also be used for iHSPCs generation. The iHSPC-derived TSP then migrates into the thymus for T lymphopoiesis

HSCs become the major source of thymopoiesis once these rare cells emerge. Reconstituting T lymphopoiesis by HSCs is an additional feasible way, but there is no robust culture method for expanding HSCs ex vivo. Generation of HSCs from pluripotent stem cells (PSCs) is a useful idea for cell therapy. Exogenous expression of hemogenic transcription factors to guide conversion of PSC-derived mesodermal cells to HSCs is a mainstream method reported by different research groups [105] (Fig. 5). Daley laboratory provides several options, such as *Hoxb4* [106], *Cdx4* [107], the combination of *HOXA9*/*ERG*/*RORA*/*SOX4*/*MYB* with *shEZH1* targeting [108], and the combination of *ERG*/*HOXA5*/*HOXA9*/*HOXA10*/*LCOR*/*RUNX1*/*SPI1* [61]. LIM-homeobox gene *Lhx2* can drive the in vitro generation of HSC-like cells from mPSCs, but the inappropriate persistence of *Lhx2* expression suppresses the developmental program at the DN stage in the thymus [109]. Terminating the *Lhx2* expression can pave the way to mature T cells from the DN stage in vivo [110], which helps to reconstitute T lymphopoiesis from PSC-derived TSP-like cells. Tan et al. found that inducing *MLL-AF4* expression promotes the generation of PSC-derived engraftable induced HSPCs (iHSPCs) with T lineage potential [111]. PSC-derived teratoma as a disorganized and spontaneous differentiation system may occasionally produce rare engraftable HSPC [112–114] (Fig. 5), but this must be optimized to control the risk of tumorigenesis. For example, large animal models, such as gene-edited immunodeficient pigs [115], can be used as containers to avoid the risk of teratoma formation or leukaemogenesis in patients, and produce sufficient engraftable iHSPCs from PSCs. Naturally, current strategies for PSC-derived iHSPC generation need to be modified by reducing the number of tumorigenesis-related TFs (such as *MYB* and *MLL-AF4*), or avoiding the formation of PSC-derived abnormal cells.

## T-cell generation meets gene editing

Several forms of adoptive T-cell transfer (ACT), such as tumor-infiltrating lymphocytes (TILs), TCR-engineered T cells (TCR-T), CAR-T, and T cell antigen coupler-engineered T cells (TAC-T) [116], have been developed for antitumor therapy [6, 117], antivirus therapy [118], and targeting cardiac fibrosis [119]. CAR-T cells have unique advantages, such as MHC-independent recognition, which can kill tumor cells without MHC-associated antigens. Compared to CAR-T, MHC-dependent TCR-T cells have the advantages of intracellular targeting, lifelong persistence, robust ability to enter the solid tumor microenvironment, and reduced cytokine release syndrome. CAR-T and TCR-T cells can both effectively eliminate tumor cells and prolong patient survival. However, the efficiency of gene editing in patient-derived primary T cells remains an obstacle, which limits the purity of antigen-specific T cells and restrict the scope of gene editing at the genomic level.

**Fig. 6:**
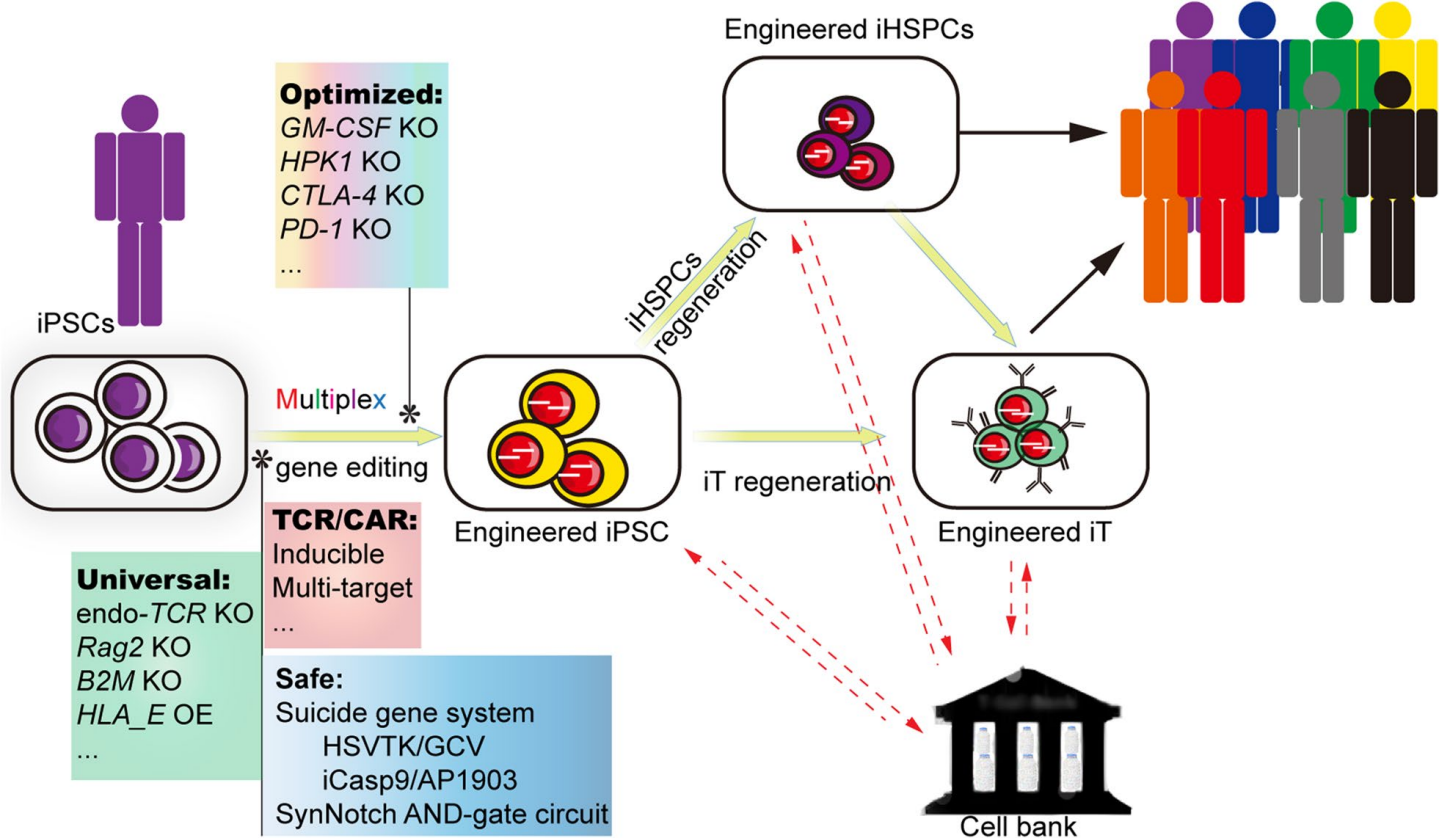
Schematic model of the combination of immunotherapy strategies and gene editing technologies in the PSC-to-T platforms. Based on the unique advantages of PSCs, we can perform gene editing to obtain engineered iPSCs for producing safe, universal and function-enhanced iT cells or iHSPCs

The advent of the PSC-to-T technique provides a scalable system that can produce large doses of gene-edited T cells in one batch, reduces substantial economic burden, increases product consistency, and easily achieves predetermined genetic engineering. Theoretically, the existing gene-editing approaches in primary T cells can be applied to PSCs more efficiently (Fig. 6). To avoid graft-versus-host-disease (GVHD), Hiroki Torikai and his colleagues made universal CD19-CAR T cells by curbing the expression of endogenous *αβ TCR* [120]. Eliminating *B2M* in transplanted cells prevents the stimulation of allogeneic T cells, and expressing *HLA-E* can help avoid allogeneic rejection by preventing host NK-mediated lysis [121]. Therefore, we can introduce multiplex gene editing in the *TCR/B2M* locus and *HLA-E* expression in PSCs for universal engineered iT generation. Moreover, inactivation of HLA-A and HLA-B, but not HLA-C, is another ideal strategy which could cover a large population [122]. T-iPSCs with *RAG2* knockout and non-T-iPSCs with transduced *TCR* can also help avoid unpredictable TCR generation [89]. It is notable that CAR, as an artificial fusion molecule, may disturb the normal pattern of T lymphopoiesis [123] and this can be overcome by constructing conditional expression cassettes at the stem cell level. Additionally, eliminating the expression of *GM-CSF* in CAR-T cells mitigates neurotoxicity and cytokine release syndrome (CRS) [124–126]. Notably, multi-target CAR-T cells are entering clinical trials [127, 128], which might help us to cope with more complex disease processes. Defects in *CTLA-4*, *PD-1*, or *HPK1* in T cells enhance T cell function [129–131]. This notion prompts the disruption of these genes to generate function-enhanced T cells from PSCs. The safety concern for engineered T cells or regenerated cells relates to the off-tumor side effects and potential tumorigenicity. These risks can be solved by employing inducible suicide gene systems, such as HSVTK/GCV and iCasp9/AP1903 [132, 133]. The synNotch AND-gate circuit is another unique strategy for reducing the adverse effects on bystander tissues [134]. According to treatment purposes, we can perform precise gene editing mentioned above in PSCs to obtain multiplex engineered PSC-iT cells. However, the current PSC-to-T platforms are inefficient, limiting the development of immune cell-based regenerative therapies. When an efficient and stable PSC-to-T platform is established, diversified immunotherapy strategies through precise gene editing technologies will quickly translated to the clinic.

## Clinical applications of PSC-derived immune cells

**Fig. 7:**
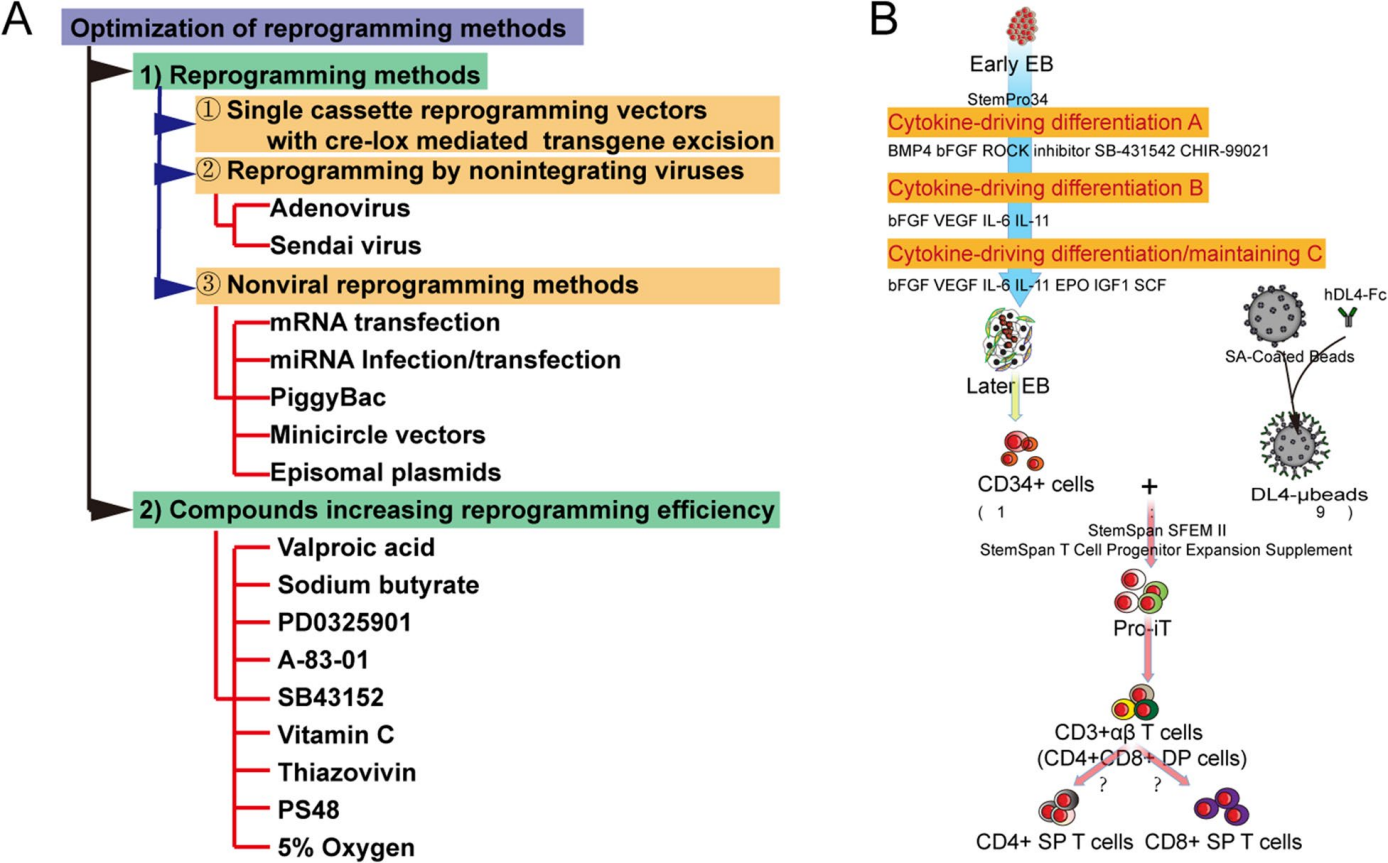
Schematic overview of the optimized reprogramming methods used in generating and maintaining iPSCs (**a**), and a stromal cell/serum-free, DL4-μbeads-based approach for progenitor T cells generation (**b**)

The PSC-derived immune cells are being quickly translated from bench to bedside. Many factors, such as safety issues and cell purity, that hinder the clinical applications of PSC-derived immune cells, are being addressed. Furthermore, various technologies have been developed to shorten the time to generate the patient-derived iPSC lines from somatic cells with adequate efficiency and safety (Fig. 7A)[135]. Unlimited sources and efficient gene-editing of iPSC show the high prospects for their clinical application and commercialization. Recently, a group reported a stromal cell/serum-free DL4-μbeads-based approach that supports the development of PSC-derived CD34^+^ cells to T lineage progenitors, which can eliminate the concerns over the safety of animal-derived substances (Fig. 7b). However, this study did not show the function or developmental progress of PSC-derived T lineage in vivo [68]. Many researchers and organizations are promoting the commercialization of iPSC-derived immune cells (Table 2). Interestingly, almost all iPSC-derived immune cell therapy products are NK cells (NK: 17/27, NK and/or T: 4/27; T: 4/27, Mac: 2/27). The reason for this may be that NK cell-mediated cytotoxicity does not require HLA-matching [136]. Several trials have demonstrated the safety of adoptive transfer of allogeneic NK cells [137]. These universal and “off-the-shelf” iPSC-derived NK cells can be produced easily. Furthermore, knocking out the *HLA* gene in iPSCs can help harvest universal iPSCs, which can subsequently be used for generation of universal iPSC- derived CAR-T cells.

**Table 2:** iPSC-derived cellular agents for immunotherapy

Organization	Product	Cell type	Tumor type	Characteristic	Phase/ClinicalTrials.gov Identifier
National Cancer Institute (NCI)	iPSC T	T	Gastrointestinal Cancers Breast Cancer Pancreatic Cancer Melanoma Lung Cancer	1) Generation of an iPSC-derived thymic organoid 2) Cancer antigen-specific T-cells	Preclinical studies/NCT03407040
Fate Therapeutics	FT819	T	B-cell Malignancies	1) CAR19 1XX placed under the control of endogenous TCR activity; 2) TCR KO	Phase 1/NCT04629729
	FT500	NK	Advanced solid tumors	–	Phase 1/NCT03841110
	FT596	NK	r/rB Lymphoma B-CLL	1) CD19 CAR 2) High-affinity 158 V, non-cleavable CD16 (hnCD16) Fc receptor 3) IL-15 receptor fusion (IL-15RF)	Phase 1/NCT04245722
	FT516	NK	r/rAML r/rB-cell lymphoma Advanced solid tumors	hnCD16 Fc receptor	Phase 1 /NCT04551885/ NCT04023071
	FT536	NK	–	1) MICA/B CAR 2) IL15RF 3) CD38 KO	Preclinical studies/-
	FT538	NK	Advanced hematologic malignancies	1) hnCD16 Fc receptor 2) IL15RF 3) CD38 KO	Phase 1/NCT04614636
	FT573	NK	Solid/hematologic malignancies	1) B7H3 CAR 2) hnCD16 Fc receptor 3) IL15RF 4) CD38 KO	Preclinical studies/-
	FT576,	NK	MM	1) BCMA CAR 2) hnCD16 Fc receptor 3) IL15RF 4) CD38 KO	Preclinical studies/-
Allogene Therapeutics + Notch Therapeutics	iPSC-AlloCAR	T/NK	NHL Leukemia MM	Generating from synthetic Engineered Thymic Niche (ETN) platform	Preclinical studies/-
Century Therapeutics	–NTY-101	NK	r/r B-cell lymphoma	1) CD19 CAR 2) expressing soluble IL-15 3) EGFR safety switch	Preclinical studies/-
	CNTY-103	NK	Recurrent glioblastoma	CD133 + EGFR CAR	Preclinical studies/-
	CNTY-102	T/NK	r/rB-cell lymphoma Other B-cell malignancies	CD19 + CD79b CAR	Preclinical studies/-
	CNTY-104	T/NK	AML	Multi-specific	Preclinical studies/-
CiRA + Takeda	iCART	T	–	–	Preclinical studies/-
Cartherics	–	NK	ovarian cancer	1) TAG72 CAR 2) Delete immune suppression gene 3) Multiple anti-cancer functionality	Preclinical studies/-
	–	T	–	CAR construct	Preclinical studies/-
Shoreline Biosciences	–	NK	–	–	Preclinical studies/-
	–	Mac	–	–	Preclinical studies/-
CellOrigin	–	NK	Hematological malignancies	–	Preclinical studies/-
	iPSC-CAR-Mac01/02/03/04	Mac	Solid Tumor	–	Preclinical studies/-
	iPSC-CAR-NK01	NK	Solid tumor	–	Preclinical studies/-
HebeCell	–	NK	–	1) Generating from 3D bioreactors 2) CAR construction	Preclinical studies/-
Neukio Biotherapeutics	–	NK	–	CAR construction	Preclinical studies/-
nuwacell	–	T/NK	–	CAR construction	Preclinical studies/-
Biotheus + iCAMUN	iPSC-CAB-NK	NK	Solid tumor	–	Preclinical studies/-
PersonGen	iPS-CAR-NK	NK	–	CAR construction	Preclinical studies/-

*NK* natural killer, *MM* multiple myeloma, *AML* acute myeloid leukemia

## Conclusions and future perspectives

The study of HSPC transplantation [138–142], as well as disorders of hematopoiesis, lymphatics, and immunity [143] has facilitated the understanding of the HSC differentiation cascade. T lineage commitment not only involves a precise transcription factor regulatory network, but also an organized thymus microenvironment [23]. Indeed, extensive research has demonstrated the feasibility of PSC-to-T [69]. To identify the T lymphopoiesis in the thymus, several single-cell transcriptional atlas of T lymphopoiesis and embryonic/adult thymus organogenesis have been established [23, 31, 144], which help us to identify the features of TSP, the interaction of thymocytes and stromal cells, and rare unconventional T subsets. Furthermore, several published scRNA-seq datasets of PSC-derived cells clearly showed the differentiation pathways and possibilities of generating physiological T-lineage cells (Table 2). More importantly, by deconstructing T lymphopoiesis in the thymus and eliminating unnecessary factors, an organized thymus-like microenvironment was reproduced in vitro for functional PSC-derived T-cell generation. The ATO co-culture system indicated the feasibility of conventional T-subset generation by constructing thymus-like niche in vitro. Defined TFs (*Runx1* and *Hoxa9*) were used to generate transplantable PSC-derived TSP. Furthermore, the improved PSC-to-T platforms through gene editing technology will likely facilitate the clinical application of PSC-T, NK and macrophage cells for cancer immunotherapy [145–152].

## Data Availability

This is not applicable for this review.

## Abbreviations

PSCs: Pluripotent stem cells
iT: Induced T
3D: three-dimensional
ATO: Artificial thymic organoid
ESCs: Embryonic stem cells
iPSCs: Induced pluripotent stem cells
CARs: Chimeric antigen receptors
HSPCs: Hematopoietic stem/progenitor cells
TSP: Thymus-seeding progenitors
HSCs: Hematopoietic stem cells
LMPP: Lymphoid-primed multipotent progenitors
CMJ: Cortico-medullary junction
ETP: Early thymic progenitors
thymic TEC: Epithelial cells
TFs: Transcription factors
FTOCs: Fetal thymic organ cultures
ATO: Artificial thymic organoid
EMP: Embryonic mesodermal progenitors
EMO: Embryonic mesodermal organoids
iHECs: Inducible hemogenic endothelial cells
scRNA-seq: Single cell RNA-seq
GVHD: Graft-versus-host-disease
ACT: T-cell transfer
TILs: Tumor-infiltrating lymphocytes
TCR-T: TCR-engineered T cells
TAC-T: T cell antigen coupler-engineered T cells
